# Biological age in diabetes and precision medicine

**DOI:** 10.18632/aging.204123

**Published:** 2022-06-06

**Authors:** Briana N. Cortez, Nadine Bahour, Cristina Aguayo-Mazzucato

**Affiliations:** 1 Beta Cell Aging Lab, Joslin Diabetes Center, Harvard Medical School, Boston, MA 02215, USA; 2 University of Texas Rio Grande Valley School of Medicine, Edinburg, TX 78539, USA

**Keywords:** biological age, diabetes mellitus, chronological age, DNA methylation, clinical biomarkers

Rates of aging vary among species and correlate with susceptibility to disease, physical and cognitive impairment, and death [1]. The mainstream use of age describes chronological age (CA), the years lived since birth. CA can directly impact biological age (BA) which specifically measures the rate of cellular decline or physiological breakdown of cells and organs within the body. While CA and BA can change at the same rate, we found that in *Diabetes mellitus*, BA is accelerated when compared to CA [2].

Given the complex nature of aging and its associated pathologies, there has not been a single method or biomarker identified to calculate BA accurately. Instead, an array of biomarkers that correlate with CA have been used to develop various algorithms. Calculating BA provides a tool to assess healthspan in age-related diseases such as Type 2 Diabetes (T2D). Over 30 million people are diagnosed with T2D in the United States, and they have a 50% higher risk of mortality. In addition, T2D correlates with an increased risk of serious health complications which decrease healthspan. The correlation between diabetes and BA can provide further insight into the increased morbidity and mortality in this disease, allow for a better understanding of aging, and lead to novel interventions.

Based on this background, we hypothesized that BA would be increased in people with *Diabetes mellitus* when compared to people without, and that this could be calculated from readily available biomarkers from routine clinical visits. We obtained data from T1D, T2D, and a prediabetic cohort, all matched to non-diabetics by age and gender. Using the Klemera and Doubal method 1 and multiple linear regression, the BA of 2459 individuals was calculated. The eight clinical biomarkers used for these calculations were: creatinine, systolic blood pressure, blood urea nitrogen, albumin, A1c, cholesterol, pulse, and diastolic blood pressure. Additionally, the phenotypic age formula with its predetermined biomarkers was also used to calculate BA. The results revealed that people with T2D had a BA 12.02 years older than non-diabetics (p<0.0001) and the BA of people with T1D was on average 16.32 years greater than non-diabetics (p<0.0001). Given the different pathophysiology of T1D and T2D, these results suggest that altered glucose metabolism leads to accelerated BA in diabetes. It is worth noting, that even though accelerated BA was observed at a population level, individuals in each group varied greatly in their speed of aging. At an individual level, a BA of 9.85 years greater than CA, carried a greater risk of mortality [2].

These results lead to important follow-up questions and implications such as the possibility of affecting the rate of BA with lifestyle and pharmacologic management of diabetes and evaluating whether accelerated aging is also observed using DNA methylation aging clocks, which are considered the gold standard to estimate BA. Future work should validate our findings showing increased biological aging in specific tissues and organs of people with T2D.

Interestingly, methylation studies and clinical biomarker algorithms used to estimate BA do not always correlate to one another, indicating that alternate algorithms may be quantifying different aspects of aging [3] such as mitochondrial dysfunction, cellular senescence, inter-cellular communication and proteostasis. A recent study [4] showed that BA measurements using clinical biomarkers exclusive to one organ system were more strongly correlated to the severity of that organ-specific disease. It is possible that in particular single organ diseases, algorithms based on organ-specific biomarkers may be a better fit to determine BA. For example, the prediction of neurocognitive disorders improves when the BA of the brain is calculated using anatomical brain and special resolution markers, including cortical thickness and surface area, subcortical volumes, and connectivity matrices [5].

In T2D, a disease that affects many organs, the optimal BA algorithm should include biomarkers from a wide range of systems which validates our use of parameters that reflect blood glucose levels, cardiovascular status, renal and hepatic function. Interestingly, we found that within our cohorts, systolic blood pressure and A1c had the strongest correlation with BA. While increased A1c ties into the pathophysiology of diabetes, the correlation to systolic blood pressure underscores the fact that cardiovascular complications are the main determinant of mortality in T2D. Therefore, using BA to assess the individual morbidity and mortality risk of any individual with T2D is a more accurate reflection of their health status than any value on its own.

The inclusion of BA into patient care may guide the field of medicine towards the precision medicine model, where treatments are based on individual patients’ characteristics. Calculating BA of individuals and their separate systems can aid physicians prioritize interventions of organs that determine accelerated aging. This will, in turn, maximize the capacity of the health system to optimize treatments and patient follow-up studies for chronic and complex diseases of aging, such as T2D.
